# Assessment of ELR, PLR, NLR and BLR Ratios during Omalizumab Treatment of Chronic Spontaneous Urticaria

**DOI:** 10.3390/jcm13154287

**Published:** 2024-07-23

**Authors:** Olga Branicka, Barbara Rymarczyk, Radosław Gawlik, Joanna Glück

**Affiliations:** Department of Internal Disease, Allergology and Clinical Immunology, Medical University of Silesia, 40-055 Katowice, Poland

**Keywords:** chronic spontaneous urticaria, biological treatment, ELR, NLR, PLR, BLR

## Abstract

**Background**: There is a need for searching for biomarkers indicating patients who will benefit the most from treatment with omalizumab for chronic spontaneous urticaria (CSU). The aim of this study was to assess whether the eosinophil/neutrophil/platelet/basophil-to-lymphocyte ratio (ELR, NLR, PLR, BLR) may predict the response to omalizumab treatment of chronic spontaneous urticaria. **Methods**: A retrospective data analysis of CSU patients treated s-c with 300 mg of omalizumab every four weeks under the drug program was carried out. NLR, ELR, PLR and BLR, DLQI, UAS-7, CRP, anti-TPO and tIgE were assessed before (V0) and after three (V3) and six months (V6) of treatment. **Results**: Among 52 patients with CSU, 21 were responders, 24 were partially responders and 6 were non-responders to treatment with 300 mg omalizumab every four weeks. An amount of 18 patients had features of type I autoallergic CSU (CSU^aiTI^) and 34 patients had autoimmunity type IIb CSU with mast cell-directed activating autoantibodies (CSU^aiTIIb^). NLR, ELR, PLR and BLR indices did not change during a six-month-course of biological treatment. Initial values of ELR and BLR were significantly correlated with the initial tIgE level and anti-TPO/IgE ratio. Initial values of NLR, ELR and BLR were significantly correlated with initial CRP. Comparisons between type I autoallergic CSU (CSU^aiTI^) and autoimmunity type IIb CSU (CSU^aiTIIb^) revealed that the absolute number and percentage of eosinophils, basophils, BLR and tIgE were significantly higher in type CSU^aiTI^ and anti-TPO and anti-TPO/IgE were significantly lower in type CSU^aiTI^. **Conclusions**: NLR, ELR, PLR and BLR do not change significantly during six months of omalizumab treatment and do not appear to be useful in predicting its efficacy.

## 1. Introduction

Chronic spontaneous urticaria (CSU) is a fluctuating inflammatory skin disorder which appears on most days for more than six weeks without an identified trigger [1]. The most affected group are women (2:1 women and men, respectively) at age 40–59 years [2]. Coexisting angioedema is reported in 33–87% of CSU cases [3]. There are no data showing racial or ethnic differences [4]. Intense itching and multiple visible hives significantly reduce the quality of life impairing daily activity, sleep, work, social activities and sexual life [5]. The exact pathogenesis of CSU is still unclear. The causes of CSU include autoimmunity type I with IgE autoantibodies to self-antigens (CSU^aiTI^, or “autoallergic” CSU) and autoimmunity type IIb with mast cell-directed activating autoantibodies (CSU^aiTIIb^). In some patients, the cause of degranulation of skin MC remains unknown (CSU^uc^). In some patients, the routine treatment with antihistamines, and treatment escalation to four-fold elevated doses, remains unsatisfactory. According to the novel results of multicenter studies, the most effective and safe second-line treatment option for these patients is biological treatment with omalizumab—a humanized, recombinant, monoclonal anti-IgE antibody. Contraindications to this type of treatment are physical triggers, parasitic infections and serious internal diseases: rheumatic diseases, thyroid disorders, inflammatory diseases and neoplasms. The variable course of the disease clearly shows that spontaneous remission is possible. However, in about 50% of cases, a more severe and prolonged form of the disease develops. The long-term prognosis is worsened by concomitant serum autoreactivity and signs of chronic inducible urticaria [6,7]. Finding the differences between responders and non-responders is the current challenge for clinical outcomes. There are data showing that platelet-to-lymphocyte ratio (PLR) as clinical markers of inflammation differ between patients with CSU and NSAID-exacerbated cutaneous disease [8]. Moreover, the same authors previously found that eosinophil-to-lymphocyte ratio (ELR) may predict oral corticosteroid dose reduction, change in quality of life and asthma control resulting from biological treatment in patients with severe asthma [9]. These observations clearly suggest that the search for predictors of treatment response is critically important and is possible. Total serum IgE level, C-reactive protein and D-dimer appear to be strong predictors of efficacy of treatment [10]. It was also suggested that increased basal IgE is found in early responders (<24 h) and increased cholesterol levels in very early responders (<24 h), and high D-dimer levels are observed in late responders (>12 weeks) [11]. The search for new salient biomarkers available in routine clinical practice is urgently needed, as they would help to determine who will benefit the most from the biological treatment. As some of these peripheral blood parameters have been proved to be useful in various allergic diseases, we raised a question if ELR, neutrophil-to-lymphocyte ratio (NLR), PLR and basophil-to-lymphocyte ratio (BLR) may predict the response to omalizumab treatment of chronic spontaneous urticaria.

## 2. Materials and Methods

This retrospective, single-center study was conducted at the University Clinical Hospital K. Gibińskiego in Katowice, Poland, at Department of Internal Disease, Allergology and Clinical Immunology. Data of patients enrolled in the omalizumab treatment program dedicated to chronic spontaneous urticaria were analyzed. Inclusion criteria were age above 18 years; documented severe chronic urticaria requires a duration of 6 weeks or more and lasting for at least one month, failure to treatment with four-fold dose of 2nd generation H1-antihistamines through four weeks in the period preceding omalizumab treatment, an urticaria activity score 7 (UAS7) ≥ 28 and Dermatology Life Quality Index (DLQI) ≥ 10 and in the case of women of child-bearing potency, declaration on effective pregnancy preventive measures. A total of 300 mg of omalizumab was given every 4 weeks.

Exclusion criteria were pregnancy or breastfeeding, contraindication to omalizumab treatment, isolated angioedema, symptomatic urticaria as a manifestation of other diseases such as anaphylaxis, neoplasms, mastocytosis or parasites, on-going therapy with immunosuppressive or anti-cancer drugs, infusions of immunoglobulins or other biological, confirmed urticarial vasculitis.

Patients were assessed before the first dose of omalizumab (V0), and then 3 months (V3) and 6 months (V6) after administration of the first dose and eventually after six months of follow-up. At the first visit, the following parameters were assessed: laboratory tests such as complete blood count, C-reactive protein (CRP), TSH, total IgE (tIgE), aminotransferase activity, creatinine and urea serum levels, UAS7, DLQI. At the V3 and V6, UAS7 and DLQI and the same laboratory tests apart from TSH, anti-TPO and tIgE were assessed. If at V3 or V6 visits UAS7 was higher than 16 points and/or DLQI was higher than 10, the treatment was interrupted because of lack of efficacy and the patients were labeled as non-responders. After V6, patients were followed up for at least 3-month intervals, and in the case of recurrence of urticarial symptoms and UAS7 score >16 points and/or DLQI >10, a next course of omalizumab treatment was introduced. This group was described as transient-responders. After follow-up period lasting up to six months, outcome of omalizumab treatment was described as full respondent if UAS7 < 16 points, DLQI < 10 points.

Moreover, all CSU patients were divided into two groups of patients with up-to-date-suggested two different immunopathogenetic types of urticaria: type I autoallergic CSU (CSU^aiTI^) and autoimmunity type IIb CSU with mast cell-directed activating autoantibodies (CSU^aiTIIb^) based on CRP value, peripheral blood eosinophil and basophil levels, anti-TPO and total IgE level [12]. CSU was defined as CSU^aiTIIb^ with the following constellation of laboratory parameters: elevated CRP, reduced peripheral blood eosinophil and basophil numbers, low or very low total IgE level, elevated IgG-anti-TPO IgG level, high IgG-anti-TPO to total IgE ratio. CSU^aiTI^ was diagnosed with normal CRP level and peripheral blood eosinophil and basophil numbers, and low IgG-anti-TPO to total IgE ratio.

### 2.1. Laboratory Tests

Blood samples were collected in a hematologic sample tube containing anticoagulant, and neutrophil, lymphocyte, eosinophil and platelet absolute counts were recorded using Sysmex XN-350 (Sysmex Europe Corporation, Norderstedt, Germany) hematology analyzer. Using these data, neutrophil, eosinophil, basophil and platelet absolute numbers were divided by the lymphocytes absolute number, and NLR ($\frac{neutrophil\;absolute\;number}{lymphocyte\;absolute\;number}$), ELR ($\frac{eosinophil\;absolute\;number}{lymphocyte\;absolute\;number}$), BLR ($\frac{basophils\;absolute\;number\;}{lymphocyte\;absolute\;number}$) and PLR ($\frac{platelet\;absolute\;number\;}{lymphocyte\;absolute\;number}$) values were calculated [9].

### 2.2. C-Reactive Protein Assay

The C-reactive protein assay was performed by latex-enhanced immunoturbidometry. Fresh human blood samples were collected aseptically, separated by standard techniques and tested within 24 h of collection. The requested sample volume for the assay was 1.0 mL. Plasma was separated within 60 min of collection. All reagents were stored at the correct temperature and used before the manufacturer’s expiry date. All controls and samples were allowed to equilibrate to room temperature prior to use, gently mixed and then diluted. A diluted solution of the test sample is then mixed with latex particles coated with monoclonal anti-CRP antibodies. CRP present in the sample forms an antigen–antibody complex with the latex particles. The light scatter, measured by a turbidometric method (biochemical analyser COBAS c 501), is proportional to the concentration of C-reactive protein in the sample. Diagnostic reagents were supplied by Roche Diagnostics. A clinical laboratory program was used to transfer the sample results to the file. The reportable range of the method is 0.6–350 mg/L (5.7–3332 nmol/L). The lowest reportable CRP result is approximately 0.3 mg/L (2.9 nmol/L). For values above the linear range of the reaction, the automatic Rerun function is activated.

### 2.3. Total IgE Assay

The total IgE assay was performed using the Cobas e801 immunochemistry analyser (Roche Diagnostics, Indianapolis, IN, USA). Venous blood samples were drawn from the antecubital vein and stored at −80 °C until analysis. The sandwich ELISA test is based on a solid phase enzyme-linked immunosorbent assay. Initial incubation leads to complex formation. The complex consists of sample antigen, biotinylated anti-IgE monoclonal antibody and rutin-labeled IgE-specific monoclonal antibody. During the next incubation, streptavidine-coated micromolecules were added to the solution and the complex was bound to the solid phase by the avidity of streptavidin and biotin. The solution was then transferred to the measurement chamber where the micromolecules were captured by the electrode surface using a magnet. Unbound material was removed with ProCell II M. The applied voltage initiates the electrochemiluminescence reaction and photon emission. This was measured with photoamplifier and read from the calibration curve. For values above the upper range of the measurement, the solution was diluted wit Diluent Universal. Recommended proportions were 1:20. The reportable range of the method is 0.2–2500 IU/mL (0.48–6000 ng/mL). The lowest reportable IgE result is approximately 0.2 IU/mL (0.24 ng/mL).

### 2.4. Anti-TPO Assay

The anti-TPO assay was performed using the COBAS e801 immunochemistry analyser. The ECLIA sandwich test is a reaction that requires two different antibodies. During the first incubation, 12 μL (e801) samples were incubated with rutin-labeled anti-TPO antibodies. During the next incubation, streptavidine-coated micromolecules and biotinylated TPO are added to the solution and anti-TPO antibodies compete with rutin-labeled anti-TPO for biotinylated TPO antigen. The solution was then transferred to the measurement chamber where the micromolecules were captured by the electrode surface using a magnet. Unbound material was removed with ProCell II M/ProCell II. The applied voltage initiates the electrochemiluminescence reaction and photon emission. This was measured with photoamplifier and read from the calibration curve. The reportable range of the method is 9–600 IU/mL. The lowest reportable anti-TPO result is <9.00 IU/mL (cobas e801).

### 2.5. Ethics

The study protocol was reviewed and approved by the Bioethics Committee at the Medical University of Silesia in Katowice (PCN/CBN/0052/KB/171/22), and was in accordance with the ethical principles for human experimentation initiated by the Declaration of Helsinki. The study was exempted from the requirement to obtain written informed consent from the patients due to the retrospective nature of the study, and need for informed consent was waived. Patient confidentiality was maintained in the analysis, and the analysis was conducted using an anonymized dataset [9].

### 2.6. Statistical Analysis

Results of CBC were expressed as absolute number and percentages. NLR, ELR, BLR and PLR, total IgE, anti-TPO and CRP were presented as median and interquartile range because of lack of normality. Results are expressed as absolute numbers and percentages for frequencies, and mean ± standard deviations (if normally distributed) or median and interquartile range (if not normally distributed). Normality was checked using the Shapiro–Wilk test. The chi-square test was used to analyze differences in nominal variables between groups. Quantitative variables were compared by Student’s *t*-test if they were normally distributed, or the Mann–Whitney *U* test and Wilcoxon signed-rank test if not normally distributed. Consecutive data were assessed with Friedman’s ANOVA test. All analyses were performed with a software package (The STATISTICA 13.3, StatSoft Polska, Kraków, Poland). *p* values less than 0.05 were considered significant [9].

## 3. Results

**Figure 1:**
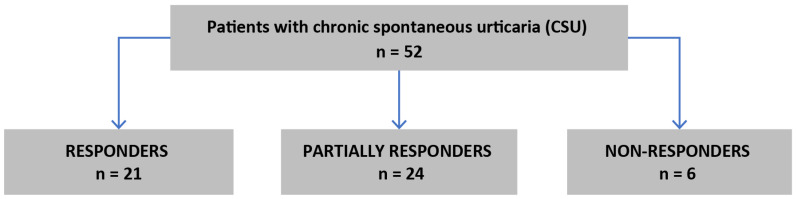
Flow chart of qualification to the study.

An amount of 52 patients (37 women) with CSU were treated with subcutaneous injections of 300 mg omalizumab every four weeks as seen in Figure 1.

Clinical characteristics of patients are shown in Table 1.

Among all patients with CSU, 21 (40%) were responders, 24 (46%) were partially responders and 6 (12%) were non-responders. NLR, ELR, PLR and BLR values did not significantly differ during the treatment course and were comparable in V0, V3 and V6 points (*p* = 0.76; *p* = 0.29; *p* = 0.14; *p* = 0.42, respectively; Friedman’s ANOVA test); see Table 2.

Initial values of ELR and BLR were significantly correlated with initial tIgE level (r_s_ = 0.53, *p* = 0.006 and r_s_ = 0.68, *p* < 0.001, respectively) and anti-TPO/IgE ratio (r_s_ = −0.44, *p* < 0.05 and r_s_ = −0.63, *p* = 0.004, respectively). Initial values of NLR, ELR and BLR were significantly correlated with initial CRP (0.23, −0.39 and −0.39, respectively). All correlations are shown in Table 3.

Initial values of NLR, ELR, PLR and BLR were comparable in all groups irrespectively of response to treatment with omalizumab. All data assessed in the three groups are shown in Table 4.

Comparisons between type I autoallergic CSU (CSU^aiTI^) and autoimmunity type IIb CSU (CSU^aiTIIb^) were noted. An amount of 18 (35%) patients had features of type I autoallergic CSU (CSU^aiTI^) and 34 (65%) patients had autoimmunity type IIb CSU with mast cell-directed activating autoantibodies (CSU^aiTIIb^). The absolute number and percentage of eosinophils, basophils, BLR and tIgE were significantly higher in the type I autoallergic CSU group, and anti-TPO and anti-TPO/IgE were significantly lower in the type I autoallergic CSU group. All data are shown in Table 5.

## 4. Discussion

In this study, authors tried to assess if NLR, ELR, PLR and BLR ratios change during omalizumab treatment and if these ratios may predict a response to the biological treatment in patients with chronic spontaneous urticaria.

We found that NLR, ELR, PLR and BLR values assessed at the beginning and after three and six months of treatment with 300 mg of omalizumab did not significantly change. Moreover, all ratios were comparable both at the beginning of treatment and after three and six months of omalizumab treatment regardless of its efficacy.

In some aspects, our results are similar to a study by Kulumbegov B. et al. who compared the neutrophil/lymphocyte ratio (NLR), eosinophil/lymphocyte ratio (ELR), platelet/lymphocyte ratio (PLR) and eosinophil/basophil ratio (EBR) in patients with chronic spontaneous urticaria (CSU) who were omalizumab sensitive and omalizumab resistant. Patients with CSU who were resistant to omalizumab treatment had a higher eosinophil/basophil ratio than those who were omalizumab sensitive [12]. However, these authors did not assess the ELR ratio. Tarkowski B. et al. also analyzed NLR and PLR as biomarkers and predictors of treatment efficacy in a group of patients with CSU treated for 6 months with omalizumab (300 mg every 4 weeks). This study did not confirm the usefulness of NLR or PLR as predictors of the response to omalizumab in CSU [13]. Platelet parameters and the neutrophil/lymphocyte ratio during omalizumab treatment in patients with severe chronic spontaneous urticaria were assessed also in another study. Ertaş R. et al. found a statistically significant decrease in the NLR (*p* = 0.018) during omalizumab treatment; however, this parameter was not useful in the prediction of response to treatment [14].

We did not find any studies in which ELR would be assessed in CSU patients treated with omalizumab. Yet a similar study was performed in a small group of patients suffering from another dermatological condition, bullous pemphigoids, treated with omalizumab, as well. The authors assessed only type 2 immunity markers such as the eosinophil-to-lymphocyte ratio (ELR), total serum IgE level, and blood eosinophil percentage. However, they did not manage to confirm that ELR may predict the response to omalizumab treatment [15].

Thus, we and other authors failed to confirm the predictive meaningfulness of peripheral blood parameters in relation to treatment with omalizumab but a higher eosinophil/basophil ratio in omalizumab-resistant patients [12]. However, recently it has been found in a large cohort of urticaria patients that the higher values of neutrophil-to-lymphocyte and platelet-to-lymphocyte ratios suggest higher odds of having chronic spontaneous urticaria rather than acute spontaneous urticaria and severe urticaria rather mild-to-moderate urticaria. Thus, these markers could be used to predict and assess severe and chronic urticaria. However, cut-off values of NLR and PLR were not given [16].

As another result of our study, we found that initial values of NLR were positively correlated with initial CRP. The NLR ratio could thus be treated as inflammatory markers, as there are a lot of data confirming that CSU is an inflammatory disease, e.g., elevated ESR, CRP, TNF-a in patients with CSU [17,18]. However, in a portion of patients, autoimmunity seems to be involved in the pathogenesis of their CSU [19,20]. In our study, initial values of ELR and BLR were significantly positively correlated with initial tIgE and negatively with initial CRP and an anti-TPO/IgE ratio.

In our further analysis of obtained results, we compared the parameters between patients with up-to-date-suggested two different immunopathogenetic types of urticaria: type I autoallergic CSU (CSU^aiTI^) and autoimmunity type IIb CSU (CSU^aiTIIb^). So far there are not unequivocal laboratory or clinical features of these two types of CSU, so we discriminated these two groups looking at a whole clinical picture in each particular patient [1]. The two groups did not differ in terms of omalizumab efficacy, but absolute number and percentage of eosinophils, basophils, BLR and tIgE were significantly higher in the type I autoallergic CSU group and anti-TPO and anti-TPO/IgE were significantly lower in the type I autoallergic CSU group. All these differences but BLR have been expected as these parameters are key laboratory features used in distinguishing between autoallergic CSU (CSU^aiTI^) and autoimmunity type IIb CSU (CSU^aiTIIb^). For us, a higher BLR ratio in autoallergic patients was an interesting observation which was probably not reported before. The multiple basophil phenotypes are observed in patients with CSU, and the response of peripheral blood basophils during treatment with omalizumab is related to the presence of autoantibodies to FcεRI on basophils [21] and to basophil phenotypes [22,23]. Thus, basophils become a novel target of research in CSU treated with omalizumab.

There are some limitations to our study. First, the size of the groups was relatively small. Another limitation is that due to the retrospective profile of the study, patients were assigned to efficacy groups after completing the study. There was no randomization. Further studies with larger numbers of patients and comparable group sizes and with randomization are indicated. In addition, further studies on omalizumab treatment of patients with chronic urticaria are indicated. It is advisable to look for indicators of long-term remission as was shown in the report by Kucharczyk et al., where it was observed that Hashimoto’s disease is a factor [24].

## 5. Conclusions

NLR, ELR, PLR and BLR do not change significantly during six months of omalizumab treatment and seem to not be useful in the prediction of its efficacy. The NLR ratio may be considered as markers of inflammation in CSU as initial values of NLR were positively correlated with initial CRP. An elevated BLR ratio in autoallergic CSU patients may be an indication for further research.

## Figures and Tables

**Table 1:** Characteristics of patients with chronic spontaneous urticaria.

	Types of CSU n = 52		
	All	CSU^aiTI^	CSU^aiTIIb^
n (%)	52	18 (35)	34 (65)
Age (years; mean ± SD)	52.0 ± 12.8	49.78 ± 13.0	53.29 ± 12.8
Male n (%)	15 (27)	8 (53)	7 (47)
Female n (%)	37 (73)	9 (24)	28 (76)
Co-morbidity n (%)			
Angioedema	23 (44)	5 (22)	18 (78)
Allergic rhinitis	8 (15)	4 (50)	4 (50)
Allergy to NSAIDs	6 (12)	3 (50)	3 (50)
Thyroid gland disease	12 (23)	2 (17)	10 (83)

CSU—chronic spontaneous urticaria; CSU^aiTI^—type I autoallergic CSU; CSU^aiTIIb^—type IIb CSU with mast cell-directed activating autoantibodies.

**Table 2:** Median values and interquartile ranges of complete blood counts and ELR, NLR, PLR and BLR ratios in patients with chronic spontaneous urticaria.

	V0 Point	V3 Point	V6 Point	*p*
Lymphocyte count, 10^3^/µL	2.14 (1.46–2.68)	2.18 (1.76–2.54)	2.26 (1.83–2.86)	NS
Lymphocytes, %	24.40 (20.80–30.40)	30.40 (24.40–35.10)	29.10 (25.05–36.0)	NS
Neutrophil count, 10^3^/µL	4.81 (3.81–6.41)	4.29 (3.20–5.67)	4.14 (3.61–5.24)	NS
Neutrophils, %	66.10 (54.20–71.40)	58.8 (53.2–64.90)	57.6 (52.75–66.75)	NS
Eosinophil count, 10^3^/µL	0.11 (0.05–0.17)	0.16 (0.10–0.25)	0.12 (0.09–0.21)	NS
Eosinophils, %	1.30 (0.60–2.20)	2.40 (1.30–3.40)	1.55 (0.90–3.05)	NS
Basophil count, 10^3^/µL	0.03 (0.001–0.04)	0.04 (0.01–0.06)	0.04 (0.02–0.06)	NS
Basophils, %	0.40 (0.10–0.60)	0.60 (0.20–0.80)	0.60 (0.20–0.80)	NS
Platelet count, 10^3^/µL	274.0 (235.5–321.5)		264.5 (226.0–322.0)	NS
ELR	0.05 (0.02–0.08)	0.07 (0.03–0.11)	0.05 (0.03–0.10)	NS
NLR	2.69 (1.78–3.34)	1.93 (1.43–2.55)	2.00 (1.50–2.66)	NS
PLR	133.57 (103.4–183.7)	124.10 (89.0–147.4)	132.30 (93.0–158.8)	NS
BLR	0.015 (0.005–0.02)	0.019 (0.007–0.02)	0.019 (0.012–0.026)	NS
CRP	3.2 (1.20–8.60)	2.4 (0.90–5.0)	3.0 (1.10–6.20)	NS
anti-TPO	10.23 (9.0–17.78)			
Total IgE	44.45 (19.85–236.50)			
anti-TPO/IgE	0.30 (0.09–0.85)			

anti-TPO—anti-thyroid peroxidase; BLR—basophil-to-lymphocytes ratio; ELR—eosinophil-to-lymphocytes ratio; PLR—platelet-to-lymphocytes ratio; NLR—neutrophil-to-lymphocytes ratio; CRP—C reactive protein; NS—non-significant.

**Table 3:** Correlation between ELR, NLR, PLR, BLR ratios and various parameters in patients with chronic spontaneous urticaria.

	NLR	ELR	PLR	BLR	tIgE	anti-TPO	anti-TPO/tIgE	CRP	DLQI	UAS7
NLR	1.000000	−0.261292	**0.581175**	0.036840	−0.070450	−0.128606	0.171882	**0.299827**	0.178170	0.064922
ELR	−0.261292	1.000000	−0.175641	**0.514202**	**0.531367**	−0.068149	**−0.441703**	**−0.397414**	0.205199	−0.002740
PLR	**0.581175**	−0.175641	1.000000	0.071887	−0.105410	−0.173240	0.141536	0.077132	0.147489	−0.054432
BLR	0.036840	**0.514202**	0.071887	1.000000	**0.684670**	−0.225203	**−0.627446**	**−0.388680**	0.195162	−0.103941

anti-TPO—anti-thyroid peroxidase; CRP—C-reactive protein; BLR—basophil-to-lymphocytes; ELR—eosinophil-to-lymphocytes ratio; PLR—platelet-to-lymphocytes ratio; NLR—neutrophil-to-lymphocytes ratio; DLQI—Dermatology Life Quality Index; UAS7—urticaria activity score 7.

**Table 4:** Median values and interquartile ranges of complete blood counts in patients with chronic urticaria depending on response to treatment.

	Responders	Partially Responders	Non-Responders	*p*
n (%)	21 (40%)	24 (46%)	6 (12%)	
Leukocytes count, 10^3^/µL	7.03 (5.80–9.06)	7.70 (6.76–10.15)	9.64 (8.17–9.94)	ns
Erythrocytes count, 10^3^/µL	4.88 (4.47–5.13)	4.62 (4.39–4.81)	4.86 (4.79–4.95)	ns
Hemoglobin count, 10^3^/µL	14.10 (13.50–14.90)	14.45 (13.40–14.95)	14.45 (13.40–15.20)	ns
Platelet count, 10^3^/µL	304 (237.0–333.0)	265.5 (234.0–292.5)	305.0 (287.0–341.0)	ns
Lymphocyte, %	25.60 (23.7–29.4)	24.15 (20.75–32.85)	22.60 (15.40–23.20)	ns
Lymphocyte count, 10^3^/µL	2.07 (1.31–2.68)	2.17 (1.47–2.70)	1.75 (1.53–2.20)	ns
Neutrophils, %	66.10 (61.20–69.70)	65.65 (51.30–70.25)	71.80 (71.70–74.60)	ns
Neutrophils count, 10^3^/µL	4.28 (3.63–6.25)	4.98 (3.95–6.07)	7.03 (6.80–7.42)	ns
Eosinophils, %	1.30 (0.70–1.80)	1.65 (0.60–2.35)	0.40 (0.40–0.90)	ns
Eosinophils count, 10^3^/µL	0.09 (0.06–0.12)	0.13 (0.05–0.23)	0.04 (0.04–0.11)	ns
Basophils, %	0.40 (0.20–0.60)	0.4 (0.15–0.55)	0.1 (0.10–0.20)	ns
Basophils count, 10^3^/µL	0.03 (0.01–0.04)	0.03 (0.02–0.04)	0.01 (0.01–0.03)	ns
PLR	137.0 (108.33–221.55)	131.13 (95.28–175.99)	155.0 (129.86–184.0)	ns
ELR	0.04 (0.03–0.07)	0.05 (0.02–0.11)	0.02 (0.01–0.06)	ns
NLR	2.58 (2.07–2.90)	2.73 (1.62–3.30)	3.18 (3.09–4.84)	ns
BLR	0.017 (0.004–0.01)	0.013 (0.006–0.02)	0.004 (0.004–0.01)	ns
anti-TPO	9.60 (9.0–16.32)	10.19 (9.0–15.72)	19.0 (10.26–30.0)	ns
Total IgE	25.10 (12.30–124.40)	104.5 (26.0–401.0)	34.9 (18.30–62.0)	ns
anti-TPO/IgE	0.41 (0.21–1.09)	0.19 (0.003–0.41)	0.58 (0.29–0.97)	ns

anti-TPO—anti-thyroid peroxidase; BLR—basophil-to-lymphocytes ratio; ELR—eosinophil-to-lymphocytes ratio; PLR—platelet-to-lymphocytes ratio; NLR—neutrophil-to-lymphocytes ratio; ns—non-significant.

**Table 5:** Median values and interquartile ranges of complete blood counts and ELR, NLR, PLR and BLR ratios in patients with chronic spontaneous urticaria, comparing two types.

	CSU^aiTI^	CSU^aiTIIb^	*p*
Lymphocyte count, 10^3^/µL	2.15 (1.56–2.68)	2.06 (1.38–2.61)	NS
Eosinophil count, 10^3^/µL	0.12 (0.08–0.17)	0.08 (0.04–0.16)	NS
Neutrophil count, 10^3^/µL	4.07 (3.63–6.31)	4.99 (3.96–6.48)	NS
Basophil count, 10^3^/µL	0.04 (0.03–0.05)	0.02 (0.01–0.04)	0.01
Platelet count, 10^3^/µL	277.0 (239.0–322.0)	272.0 (229.5–312.5)	NS
ELR	0.06 (0.04–0.09)	0.04 (0.02–0.08)	NS
NLR	2.33 (1.48–2.91)	2.82 (2.03–3.36)	NS
PLR	131.2 (94.2–183.7)	135.3 (107.8–182.8)	NS
BLR	0.02 (0.02–0.03)	0.01 (0.0–0.02)	0.01
Total IgE	423.0 (206.0–971.0)	34.40 (18.0–63.60)	0.0001
Anti-TPO	9.0 (9.0–14.0)	11.05 (9.0–24.0)	NS
anti-TPO/tIgE	0.02 (0.01–0.04)	0.41 (0.21–0.98)	0.00002

CSU—chronic spontaneous urticaria; CSU^aiTI^—type I autoallergic CSU; CSU^aiTIIb^—type IIb CSU with mast cell-directed activating autoantibodies; BLR—basophil-to-lymphocytes ratio; ELR—eosinophil-to-lymphocytes ratio; PLR—platelet-to-lymphocytes ratio; NLR—neutrophil-to-lymphocytes ratio; NS—non-significant.

## Data Availability

All data generated or analyzed during this study are included in this article. Further enquiries can be directed to the corresponding author.

## References

[1] Zuberbier T., Abdul Latiff A.H., Abuzakouk M., Aquilina S., Asero R., Baker D., Ballmer-Weber B., Bangert C., Ben-Shoshan M., Bernstein J.A. (2022). The international EAACI/GA^2^LEN/EuroGuiDerm/APAAACI guideline for the definition, classification, diagnosis, and management of urticaria. Allergy.

[2] Wertenteil S., Strunk A., Garg A. (2019). Prevalence estimates for chronic urticaria in the United States: A sex- and age-adjusted population analysis. J. Am. Acad. Dermatol..

[3] Guo C., Saltoun C. (2019). Urticaria and angioedema. Allergy Asthma Proc..

[4] Sanchez-Borges M., Ansotegui I.J., Baiardini I., Bernstein J., Canonica G.W., Ebisawa M., Gomez M., Gonzalez-Diaz S.N., Martin B., Morais-Almeida M. (2021). The challenges of chronic urticaria part 1: Epidemiology, immunopathogenesis, comorbidities, quality of life, and management. World Allergy Organ. J..

[5] Goncalo M., Gimenez-Arnau A., Al-Ahmad M., Ben-Shoshan M., Bernstein J.A., Ensina L.F., Fomina D., Galvàn C.A., Godse K., Grattan C. (2021). The global burden of chronic urticaria for the patient and society. Br. J. Dermatol..

[6] Curto-Barredo L., Archilla L.R., Vives G.R., Pujol R.M., Gimenez-Arnau A.M. (2018). Clinical features of chronic spontaneous urticaria that predict disease prognosis and refractoriness to standard treatment. Acta Derm. Venereol..

[7] Genentech (2021). Xolair Prescribing Information.

[8] Branicka O., Rogala B., Glück J. (2020). Eosinophil/Neutrophil/Platelet-to-lymphocyte ratios in various types of immediate hypersensitivity to NSAIDS: A preliminary Study. Int. Arch. Allergy Immunol..

[9] Branicka O., Gawlik R., Glück J. (2024). Eosinophil to lymphocyte ratio may predict OCS reduction and change in quality of life (AQLQ) resulting from asthma biological treatment. Immunopharmacol. Immunotoxicol..

[10] Fok J.S., Kolkhir P., Church M.K., Maurer M. (2021). Predictors of treatment response in chronic spontaneous urticaria. Allergy.

[11] Grieco T., Dies L., Sernicola A., Chello C., Gagliostro N., Carnicelli G., Paolino G. (2020). Potential clinical and serological predictors of chronic spontaneous urticaria relapse in patients under omalizumab treatment. Immunotherapy.

[12] Kulumbegov B., Chikovani T., Gotua M., Kikodze N., Magen E. (2023). Interleukin-33, endothelin-1, and inflammatory parameters in chronic spontaneous urticaria. Allergy Asthma Proc..

[13] Tarkowski B., Ławniczak J., Tomaszewska K., Kurowski M., Zalewska-Janowska A. (2023). Chronic Urticaria Treatment with Omalizumab-Verification of NLR, PLR, SIRI and SII as Biomarkers and Predictors of Treatment Efficacy. J. Clin. Med..

[14] Ertaş R., Özyurt K., Karakükçü Ç., Akkuş M.R., Özlü E., Avcı A., Atasoy M. (2018). Evaluation of platelet parameters and neutrophil/lymphocyte ratio during omalizumab treatment in patients with severe chronic spontaneous urticaria. Turk. J. Med. Sci..

[15] Akin G., Avci C., Akarsu S. (2024). The Significance of Eosinophil-to-Lymphocyte Ratio in Predicting Response to Omalizumab Treatment in Patients with Bullous Pemphigoid: A Case Series. Indian J. Dermatol..

[16] Weissmann S., Burrack N., Golan–Tripto I., Horev A. (2024). Increased Neutrophil–Lymphocyte Ratio and Platelet-Lymphocyte Ratio in Chronic and Severe Urticaria. Acta Derm. Venereol..

[17] Can Bostan O., Damadoglu E., Sarac B.E., Kilic B., Sahiner U.M., Karaaslan C., Karakaya G., Kalyoncu A.F. (2023). Cytokine Profiles of Chronic Urticaria Patients and The Effect of Omalizumab Treatment. Dermatol. Pract. Concept..

[18] Kolkhir P., Altrichter S., Hawro T., Maurer M. (2018). C-reactive protein is linked to disease activity, impact, and response to treatment in patients with chronic spontaneous urticaria. Allergy Eur. J. Allergy Clin. Immunol..

[19] Puxeddu I., Petrelli F., Angelotti F., Croia C., Migliorini P. (2019). Biomarkers In Chronic Spontaneous Urticaria: Current Targets And Clinical Implications. J. Asthma Allergy.

[20] Asero R., Ferrer M., Kocaturk E., Maurer M. (2023). Chronic Spontaneous Urticaria: The Role and Relevance of Autoreactivity, Autoimmunity, and Autoallergy. J. Allergy Clin. Immunol. Pract..

[21] Oda Y., Fukunaga A., Washio K., Imamura S., Mizuno M., Hatakeyama M., Ogura K., Nishigori C. (2021). Improved FcεRI-Mediated CD203c Basophil Responsiveness Reflects Rapid Responses to Omalizumab in Chronic Spontaneous Urticaria. J. Allergy Clin. Immunol. Pract..

[22] Johal K.J., Chichester K.L., Oliver E.T., Devine K.C., Bieneman A.P., Schroeder J.T., MacGlashan D.W., Saini S.S. (2021). The efficacy of omalizumab treatment in chronic spontaneous urticaria is associated with basophil phenotypes. J. Allergy Clin. Immunol..

[23] MacGlashan D., Saini S., Schroeder J.T. (2021). Response of peripheral blood basophils in subjects with chronic spontaneous urticaria during treatment with omalizumab. J. Allergy Clin. Immunol..

[24] Kucharczyk A., Marczyk K., Kucharczyk B., Plisko R., Perkowska J., Owczarek W., Jahnz-Różyk K. (2024). Predicting relapse in chronic spontaneous urticaria: A retrospective cohort study evaluating omalizumab withdrawal regiments. Allergy.

